# Change in Clinical Management of Localized Prostate Cancer Patients at a Tertiary Medical Center as a Result of SARS‐CoV‐2 (COVID‐19)

**DOI:** 10.1155/proc/9996270

**Published:** 2026-01-15

**Authors:** Alexander Yaney, Jonathan E. Schoenhals, Yevgeniya Gokun, Andrew Stevens, Jack Wang, Jessica Aduwo, Ahmad Shabsigh, Shawn Dason, Akshay Sood, Evan Thomas, Jacob Eckstein, Russell Palm, Rebekah Young, Dayssy Alexandra Diaz Pardo, Douglas Martin, Shang-Jui Wang

**Affiliations:** ^1^ Department of Radiation Oncology, The Ohio State University James Cancer Center, Columbus, Ohio, USA; ^2^ Secondary Data Core, Center for Biostatistics, The Ohio State University Wexner School of Medicine, Columbus, Ohio, USA; ^3^ The Ohio State University College of Medicine, Columbus, Ohio, USA, osu.edu; ^4^ Department of Urology, The Ohio State University Wexner Medical Center, Columbus, Ohio, USA, osu.edu

**Keywords:** ADT, COVID-19, global pandemic, prostate cancer, radiation therapy, radical prostatectomy

## Abstract

**Background:**

At the height of the COVID‐19 pandemic, healthcare utilization among cancer patients, including those with prostate cancer, was limited due to inadequate healthcare resources and concern for disease transmission among patients and medical personnel. While publications with treatment recommendations during the pandemic exist, there is limited real‐life data comparing prostate cancer management pre‐COVID to during the pandemic.

**Objective:**

The primary aim of this study is to determine the effect of the COVID‐19 pandemic on prostate cancer management at a tertiary medical center by comparing patients receiving treatment preceding the pandemic to those receiving treatment during the height of the pandemic.

**Methods:**

Prostate cancer patients treated with definitive intent (surgery, definitive radiation (RT), or salvage RT) in 2019–2020 were retrospectively reviewed. Analysis was performed with the following timeframes: pre‐COVID as 1/3/19 to 2/28/20 and COVID as 3/1/20 to 9/30/20. Time‐to‐treatment (TT) for surgery and definitive RT was defined from the date of diagnosis (biopsy or decision to treat if active surveillance) to the date of surgery or RT start, respectively. TT for salvage RT was defined as the date of PSA failure to RT start. Descriptive statistics such as medians and interquartile ranges (IQRs) were reported for continuous variables, while frequency counts and percents were reported for categorical variables. Chi‐squared tests (Fisher’s exact when appropriate) along with Wilcoxon rank sum tests were used to compare two timeframes. Two adjusted binary logistic regressions assessed the associations of NCCN risk groups, as well as grade group (GG), on the outcome of receipt of treatment during COVID compared to those in pre‐COVID timeframes.

**Results:**

This study sample included 565 prostate cancer patients treated with surgery (*n* = 303), definitive RT (*n* = 151), or salvage RT (*n* = 111). There was a statistically significant difference in TT by timeframe for all patients (median 111 days pre‐COVID v 126.5 days COVID, *p* = 0.007). For patients who received definitive or salvage RT with ADT, there were significant differences in time from ADT initiation to treatment start between pre‐COVID vs. COVID (definitive RT: median 78 days v 147 days, *p* = 0.001; salvage RT: median 67 days v 84 days, *p* = 0.004). A significantly higher proportion of patients received ADT prior to surgery in the COVID cohort compared to those in pre‐COVID (9.8% v 0.5%, *p* < 0.001). Patients with clinically more aggressive prostate cancer had higher odds of being treated in the COVID timeframe (HR or VHR, adjusted OR [aOR] 1.62, 95% CI: 1.02–2.55).

**Conclusions:**

The COVID‐19 pandemic changed prostate cancer management in various ways including longer TT, prolonged time from ADT initiation to RT, increased utilization of ADT for patients planned for surgery, and prioritizing treatment for higher‐risk disease. Analysis of treatment outcomes for these patients may direct action in future global pandemics.

## 1. Introduction

SARS‐CoV‐2 (COVID‐19) is a novel coronavirus that is disseminated throughout the world and continues to persist as well as pose a substantial health risk to many [1]. As of October 6, 2025 there have been at least 7.1 million deaths and 778 million infections globally [1]. Multiple risk factors are associated with an increased risk of developing severe infections to and complications from COVID‐19, including having a cancer diagnosis [2–5]. Due to the widespread and severe nature of these infections stressing many already resource‐limited healthcare systems and in an effort to decrease transmission of the virus to both patients and medical personnel, many professional groups and hospital systems stressed the importance of limiting healthcare utilization among cancer patients, including those with prostate cancer [6–13]. This extended to recommendations against performing routine screening prostate specific antigen (PSA) testing for asymptomatic patients [14, 15]. However, the impact this altered management for prostate cancer patients may have with regard to long‐term cancer‐specific outcomes is controversial and likely depends on risk category and clinical staging [8, 16–19].

While numerous publications regarding treatment recommendations during the COVID‐19 pandemic exist, there is limited actual data comparing pre‐COVID‐19 prostate cancer management to management during the pandemic. Some studies that do exist found either an increased utilization of radiotherapy and surgery or no changes in either modality for treatment of prostate cancer during the pandemic compared to prior [20–22]. Another study pointed toward a decreased utilization of radiation (RT) for prostate cancer initially, followed by a significant rebound [23]. Other studies showed differing rates of prostatectomy, including based on higher‐risk pathology or even based on race [24, 25]. The primary aim of this study is to determine the effect of the COVID‐19 pandemic on the management of prostate cancer patients at a large, tertiary medical center by comparing patients receiving treatment immediately preceding the pandemic to those receiving treatment during the pandemic.

## 2. Materials and Methods

After receiving institutional review board approval, patients with prostate cancer treated definitively with either surgery, RT, or salvage RT at our institution from 2019 to 2020 were included in this study and were retrospectively reviewed. Patients were excluded if they had metastatic disease found prior to treatment. Two timeframes were used to determine pre‐COVID (1/3/19–2/28/20) and COVID (3/1/20–9/30/20) periods based on the date of treatment receipt.

Race was categorized as white, black, or other. ADT was categorized as neither indicated nor received, indicated only, received only, or both indicated and received. Age was determined based on the start of treatment. We defined the time‐to‐treatment (TT) for intervention as the date of diagnosis via biopsy or decision to treat if in active surveillance to the date of intervention. TT for salvage RT was defined as the date of PSA failure to the start of RT. PSA values were obtained at the time of diagnosis or biochemical recurrence. Risk stratification was determined at the time of initial diagnosis based on NCCN guidelines for very low risk (VLR), low risk (LR), favorable intermediate risk (FIR), unfavorable intermediate risk (UIR), high risk (HR), and very high risk (VHR). Grade group (GG) was determined based on pathologic scores as determined by the pathologist and ranged from 1 to 5.

Patients who were treated with surgery underwent radical prostatectomy with or without pelvic lymph node dissection. Patients treated with definitive or salvage RT received stereotactic body radiation (SBRT) or external beam radiation (EBRT), with or without the addition of low‐dose rate brachytherapy. Targets for RT included the prostate with or without pelvic lymph nodes for patients who were treated with definitive RT, and the prostatic fossa with or without the pelvic lymph nodes in patients who received salvage RT.

### 2.1. Statistical Analysis

Descriptive statistics are displayed as frequencies and percents for categorical variables and medians and interquartile ranges (IQRs) for continuous variables. Comparison of variables related to treatments between the two timeframe groups was performed using chi‐squared tests (or Fisher’s exact tests when appropriate) for categorical variables, while Wilcoxon rank sum tests were used for continuous variables.

Crude binary logistic regressions modeled the receipt of treatment during COVID vs pre‐COVID timeframes between surgery and definitive RT modalities. Two separate multivariable models were ran: one was adjusted for age, race (white vs non‐white), PSA, and GG (1–3 vs 4–5), while another one was adjusted for age, race (white vs non‐white), PSA, and risk stratification (VLR/LR/FIR/UIR or “low risk” vs HR/VHR or “high risk”). Both GG and risk stratification were not included in the same model together due to multicollinearity between two covariates measured by variance inflation factors (VIFs of 4). Race, GG, and risk stratification variables were dichotomized accordingly to avoid comparing categories with very small sample sizes in these models.

Additional bivariate analysis included comparing treatment characteristics between two timeframes stratified by risk group (“low‐risk disease” or “high‐risk disease”) with the use of Wilcoxon rank sum test for continuous variables and chi‐squared test (or Fisher’s exact when appropriate) for categorical ones.

These analyses were performed using SAS v 9.4 (SAS Institute; Cary, NC; https://www.sas.com). Statistical significance was defined as two‐sided alpha < 0.05.

## 3. Results

### 3.1. Patient Characteristics

Overall, 565 patients were identified who met our inclusion criteria—303 patients received surgery, 151 received definitive RT, and 111 received salvage RT. Of those, the median age was 64 for all patients (Table 1). The majority of patients were white (80.9%), and 69.2% of patients received treatment in the pre‐COVID timeframe. Of the entire study cohort, 64.8% of patients had GG of 1–3. For the patients who received RT, most patients received non‐SBRT EBRT, 99 (65.6%) in the definitive setting and 111 (100.0%) in the salvage setting. The pelvis was included in 73 patients (48.3%) who received definitive RT versus 70 patients (63.1%) receiving salvage RT.

**Table 1:** Descriptive statistics between three treatment plans among prostate cancer patients (*N* = 565).

Variable	Total (*N* = 565)	Surgery patients (*n* = 303)		RT patients (*n* = 151)		Salvage patients (*n* = 111)	
Timeframe		Pre‐COVID (*n* = 221)	COVID (*n* = 82)	Pre‐COVID (*n* = 99)	COVID (*n* = 52)	Pre‐COVID (*n* = 71)	COVID (*n* = 40)
Age (years) Median (*Q*_1_, *Q*_3_) Range	64 (59, 69) 45–85	64 (59, 69) 45–84	62.5 (58–67) 47–79	68 (61, 72) 47–84	67 (61, 73) 51–85	64 (58, 69) 47–81	62 (58.5, 67.5) 50–74
Race White Black Other/Missing	457 (80.88%) 91 (16.11%) 17 (3.01%)	187 (84.62%) 29 (13.12%) 5 (2.26%)	68 (82.93%) 13 (15.85%) 1 (1.22%)	70 (70.71%) 23 (23.23%) 6 (6.06%)	36 (69.23%) 16 (30.77%) 0 (0.00%)	64 (90.14%) 4 (5.63%) 3 (4.23%)	32 (80.00%) 6 (15.00%) 2 (5.00%)
Grade Group 1 2 3 4 5 Missing	52 (9.20%) 207 (36.64%) 107 (18.94%) 120 (21.24%) 74 (13.10%) 5 (0.88%)	26 (11.76%) 85 (38.46%) 52 (23.53%) 39 (17.65%) 19 (8.60%) 0 (0.00%)	7 (8.54%) 33 (40.24%) 13 (15.85%) 19 (23.17%) 10 (12.20%) 0 (0.00%)	11 (11.11%) 38 (38.38%) 17 (17.17%) 19 (19.19%) 13 (13.13%) 1 (1.01%)	1 (1.92%) 18 (34.62%) 5 (9.62%) 17 (32.69%) 10 (19.23%) 1 (1.92%)	5 (7.04%) 21 (29.58%) 17 (23.94%) 11 (15.49%) 14 (19.72%) 3 (4.23%)	2 (5.00%) 12 (30.00%) 3 (7.50%) 15 (37.50%) 8 (20.00%) 0 (0.00%)
Risk Group VLR LR FIR UIR HR VHR Missing	4 (0.71%) 36 (6.37%) 146 (25.84%) 165 (29.20%) 124 (21.95%) 85 (15.04%) 5 (0.88%)	4 (1.81%) 16 (7.24%) 67 (30.32%) 67 (30.32%) 49 (22.17%) 18 (8.14%) 0 (0.00%)	0 (0.00%) 6 (7.32%) 21 (25.61%) 23 (28.05%) 15 (18.29%) 17 (20.73%) 0 (0.00%)	0 (0.00%) 6 (6.06%) 31 (31.31%) 28 (28.28%) 16 (16.16%) 17 (17.17%) 1 (1.01%)	0 (0.00%) 1 (1.92%) 13 (25.00%) 11 (21.15%) 17 (32.69%) 10 (19.23%) 0 (0.00%)	0 (0.00%) 5 (7.04%) 8 (11.27%) 28 (39.44%) 17 (23.94%) 10 (14.08%) 3 (4.23%)	0 (0.00%) 2 (5.00%) 6 (15.00%) 8 (20.00%) 10 (25.00%) 13 (32.50%) 1 (2.50%)
PSA Median (*Q*_1_, *Q*_3_) Range	5.93 (4.10, 10.00) 0.05–268.00	6.98 (5.09–10.80) 1.46–83.04	6.47 (5.02, 12.16) 1.39–268.00	7.67 (5.40, 13.40) 1.63–97.79	6.77 (4.97, 10.58) 2.90–136.82	0.16 (0.11, 0.40) 0.05–3.93	0.21 (0.14, 0.48) 0.06–13.10
RT Type SBRT EBRT Brachy + EBRT		N/A	N/A	31 (31.31%) 65 (65.66%) 3 (3.03%)	12 (23.08%) 34 (65.38%) 6 (11.54%)	0 (0.00%) 71 (100.00%) 0 (0.00%)	0 (0.00%) 40 (100.00%) 0 (0.00%)
RT Volume Prostate only Prostate and pelvis Fossa only Fossa and nodes		N/A	N/A	54 (54.55%) 45 (45.45%) 0 (0.00%) 0 (0.00%)	24 (46.15%) 28 (53.85%) 0 (0.00%) 0 (0.00%)	0 (0.00%) 0 (0.00%) 31 (43.66%) 40 (56.34%)	0 (0.00%) 0 (0.00%) 10 (25.00%) 30 (75.00%)

### 3.2. Differences in Treatment Modality and TT Between Pre‐COVID and COVID

We first sought to identify any institutional changes based on treatment modality in the two timeframes (Table 2). There did not appear to be any significant differences between utilization of treatment modality with surgery or definitive RT in the pre‐COVID or COVID timeframes (*p* = 0.105). There was a significant difference in TT for all patients regardless of modality in the pre‐COVID and COVID timeframes, with a median of 111 vs. 126.5 days, respectively (*p* = 0.007). When looking at surgery individually, there was not a significant difference in TT between pre‐COVID and COVID timeframes (median time 102 days vs. 90.5 days, *p* = 0.137). However, among the patients receiving definitive RT, there was significantly longer TT in the COVID compared to that in pre‐COVID timeframes (median 204.5 vs. 145 days, *p* < 0.001); this was seen among the patients receiving salvage RT as well (median 143 vs. 112 days, *p* = 0.004).

**Table 2:** Comparing clinical characteristics between pre‐COVID and COVID timeframes.

Variable	Pre COVID	COVID	*p*‐value
Time from diagnosis to treatment (days)^1^ Median (*Q*_1_, *Q*_3_) Range	111 (83, 155) 26–745	126.5 (89, 199) 0–369	**0.007**
Treatment Surgery Definitive RT	221 (69.06%) 99 (30.94%)	82 (61.19%) 52 (38.81%)	0.105
ADT receipt prior to surgery Yes No	1 (0.45%) 220 (99.55%)	8 (9.76%) 74 (90.24%)	**< 0.001**
Time from diagnosis to surgery (days) Median (*Q*_1_, *Q*_3_) Range	102 (76, 134) 26–512	90.5 (62, 130) 0–349	0.137
Time from diagnosis to definitive RT (days) Median (*Q*_1_, *Q*_3_) Range	145 (103, 195) 28–745	204.5 (159, 265.5) 72–369	**< 0.001**
Time from diagnosis to salvage RT (days) Median (*Q*_1_, *Q*_3_) Range	112 (90, 150) 34–336	143 (112, 194.5) 60–286	**0.004**
Time from ADT to definitive RT (days) Median (*Q*_1_, *Q*_3_) Range	78 (59.5, 127) 30–350	147 (83, 199) 48–341	**0.001**
Time from ADT to salvage RT (days) Median (*Q*_1_, *Q*_3_) Range	67 (51, 91) ‐1, 304	84 (69, 117) 21, 265	**0.016**

*Note:* The bold *p* values are statistically significant (*p* value < 0.05).

^1^Measured among entire cohort (surgery, definitive RT, and salvage RT).

### 3.3. Treatment in Relation to ADT Initiation

We evaluated whether there was any relationship between treatment modality and ADT initiation, as there may have been delayed and/or limited operating room times for surgery and treatment times for RT during the COVID timeframe. There was a significant difference in number of patients who received androgen deprivation therapy (ADT) prior to getting surgery among the 303 surgery patients. Among this surgery cohort, higher proportion received ADT prior to surgery during COVID compared to that of pre‐COVID timeframe (9.8% vs 0.5%, *p* < 0.001). When further stratifying based on LR (VLR/LR/FIR/UIR) vs HR (HR/VHR) patients, we found that a higher proportion of HR patients (6 of 32 patients, 18.75%) received ADT prior to surgery during COVID compared with that of LR patients (2 of 50 patients, 4%) (Supporting Information 2).

There was also a significant difference in time from initiation of ADT to treatment in patients undergoing definitive RT, with median times of 78 days in pre‐COVID vs. 147 days in COVID (*p*‐value = 0.001). In addition, patients who were receiving salvage RT had a significant difference in the time from initiation of ADT to starting treatment, with a median of 67 days in pre‐COVID compared to 84 days in COVID (*p*‐value = 0.016).

### 3.4. Treatment Modality and Patient Factor Differences Between Pre‐COVID and COVID Timeframes

As RT treatments tend to be more flexible than operating room times, we hypothesized that patients would be more likely to get RT as opposed to surgery during COVID. To our surprise, both crude and adjusted models showed that treatment modality was not significantly associated with its receipt in COVID vs pre‐COVID timeframes. Age, race, and PSA were also not significant predictors in the adjusted models.

We also hypothesized that patients who had a higher‐risk disease, either by GG or NCCN risk stratification, would be more likely to receive primary treatment (surgery or definitive RT) during COVID as a strategy to prioritize treatment of more aggressive disease during times of limited resources. High‐risk (HR or VHR) patients were at 62% higher odds of receiving treatment during COVID compared to LR (VLR/LR/FIR/UIR) patients (aOR: 1.62, 95% CI: 1.02–2.55; Table 3). Also, in a separate adjusted model, patients with GG of 4 or 5 were at 76% higher odds of receiving treatment during COVID compared to patients with GG of 1 through 3 (aOR: 1.76, 95% CI: 1.11–2.78, Supporting Information 1).

**Table 3:** Multivariable analysis of treatment modality and patient factors for COVID compared to that of pre‐COVID.

Variable	Crude OR (95% CI), *p*‐value	Adjusted OR (95% CI), *p*‐value^1^
Treatment Surgery Definitive RT	Ref 1.42 (0.93–2.16), *p* = 0.105	Ref 1.51 (0.96–2.36), *p* = 0.075
Age	0.99 (0.97–1.02), *p* = 0.646	0.98 (0.95–1.01), *p* = 0.177
Race White Non‐White (Black, Other)	Ref 1.14 (0.69–1.87), *p* = 0.610	Ref 0.95 (0.57–1.60), *p* = 0.844
PSA	1.01 (1.00–1.02), *p* = 0.069	1.01 (1.00–1.02), *p* = 0.299
Risk Group Low‐Risk Disease^2^ High‐Risk Disease^3^	Ref 1.72 (1.14–2.61), *p* = 0.010	Ref 1.62 (1.02–2.55), *p*=**0.040**

*Note:* The bold *p* values are statistically significant (*p* value < 0.05).

Abbreviations: CI = confidence interval; OR = odds ratio.

^1^Adjusted for treatment, age, race, PSA, and risk group.

^2^Defined as VLR, LR, FIR, and UIR.

^3^Defined as HR and VHR.

## 4. Discussion

In this study, we identified 565 prostate cancer patients who received treatment at our institution between January 2019 through September 2020. We sought to determine if there were any differences in treatment approach and TT in prostate cancer patients as determined by time of diagnosis, with March 1, 2020 defined as the start of the COVID‐19 pandemic, ending September 30, 2020 as clinical operations began to normalize. We demonstrate in this study that TT comparing pre‐COVID to COVID timeframes was significant in all patients, with median times of 111 and 126.5 days, respectively (*p*‐value = 0.007; Table 2). We found a statistically significant difference in TT for definitive and salvage RT during COVID compared to that of pre‐COVID timeframes (median 204.5 vs. 145 days, *p* < 0.001 and median 143 vs. 112 days, *p* = 0.004, respectively). The proportion of patients receiving either definitive RT or surgery between the two timeframes was not significant (*p* = 0.105; Table 2). We did not find any differences with regard to race and definitive treatment, in contrast to what was seen in a study published on patients treated with prostatectomy during the pandemic (Tables 1 and 3) [24]. In addition, we found that patients receiving ADT in the definitive and salvage RT settings appeared to have significantly longer time from ADT initiation to starting treatment during COVID (definitive RT: median 78 days vs. 147 days, *p* = 0.001, and salvage RT: median 67 days vs. 84 days, *p* = 0.016, in the pre‐COVID vs COVID timeframes, respectively; Table 2). In patients planned to undergo surgery, a significantly higher proportion of patients were placed on ADT prior to surgery during COVID compared to those in pre‐COVID (*p*‐value < 0.001; Table 2). Lastly, patients with higher‐risk disease (HR and VHR) had higher odds of being treated during COVID (aOR: 1.62, 95% CI: 1.02–2.55; Table 3).

As addressed previously, there are limited studies discussing the differences in utilization of surgery and RT during the COVID‐19 pandemic. One study by Pepe et al. demonstrated increased utilization of both prostatectomy and RT compared with active surveillance during COVID‐19 [20]. Another study performed in Sweden demonstrated decreased registrations of prostate cancer during the COVID‐19 pandemic, most pronounced in patients aged 70 or older. There did not appear to be any decrease in the total number of radical prostatectomies or EBRT treatments [21]. An Australian experience detailing courses of RT before and during COVID did demonstrate an initial drop in treatments during COVID‐19, which quickly rebounded after a few months [23]. One study based out of Brazil demonstrated no changes in diagnosis nor treatment during COVID [22]. Lastly, one multi‐institutional study out of Pennsylvania noted decreased biopsy and prostatectomy volumes [25]. Of note, none of these studies investigated the multitude of changes in patterns of clinical care. In our institutional experience, we did see an increased TT in the COVID timeframe in all patients. While this was statistically significant, given the indolent nature of the disease and overall general good oncologic outcomes with monitoring such as active surveillance, it is unclear whether an increase in 15 days of median TT has a significant clinical impact on outcome for patients [26]. The TT delay was longer with regard to RT therapy, with an increase in 59.5 days of median TT for definitive RT and 31 days for salvage RT, but again it is unclear if this is of clinical significance. Of note, at our cancer institution, apart from a 4‐week period in April 2020 with a complete halt in prostatectomy surgery, there were no other significant delays in surgery, in contrast to what may have occurred in other parts of the country.

One strategy to delay definitive treatment for prostate cancer in times of limited resources is through the use of neoadjuvant ADT. Of note, we did see a difference in time from initiation of ADT to starting RT, either in the upfront or salvage settings (Table 2). This difference appeared to be more drastic in the upfront setting, with median times of 78 days vs 147 days in the pre‐COVID and COVID timeframes, respectively. Neoadjuvant ADT prior to RT has been historically administered for IR and HR prostate cancer. More recent data in the form of two major studies, Ottawa 0101 and RTOG 9413, demonstrated that, when timing short‐term androgen deprivation either in the neoadjuvant versus concurrent setting, there does not appear to be any differences in outcomes with regard to PFS or OS [27, 28]. An additional NCDB study suggests similar findings, specifically highlighting no detriment to overall survival when RT was initiated within 6 months of ADT [29]. However, one meta‐analysis encompassing 12 randomized trials demonstrated that for prostate‐only RT, concurrent/adjuvant RT was associated with improved metastasis‐free survival, prostate cancer‐specific mortality, and overall survival [30]. The optimal timing of ADT and definitive RT for treatment of localized prostate cancer remains controversial, but in the setting of limited healthcare resources in which RT needs to be delayed, prolonging time of ADT to start of RT within a reasonable time period (i.e., 6 months), while not evidence‐based, is likely a safe strategy and unlikely to compromise treatment outcomes for patients.

In addition to increased times from ADT to starting RT, we also saw increased utilization of ADT prior to surgery (one patient in pre‐COVID vs eight patients in COVID). When looking at HR patients (HR/VHR) in our cohort, we found that there were six patients who were placed on ADT prior to surgery during COVID, compared with one patient pre‐COVID (*p*‐value = 0.004; Supporting Table 2). Given the uncertainty on the impact COVID might have with regard to feasibility of timely surgery, this approach was adopted for some patients at our institution to mitigate any detrimental impact of disease progression or metastasis from potential delay to surgery. The role of neoadjuvant ADT prior to surgical intervention is unclear, with a large NCDB study demonstrating association of neoadjuvant ADT with a decreased positive margin in patients with low‐ and intermediate‐risk prostate cancer [31]. A phase 3 randomized study investigated the use of chemotherapy in addition to ADT for HR prostate cancer patients in the neoadjuvant setting and did not demonstrate any improvement in 3‐ or 5‐year biochemical progression free survival when compared to surgery alone [32]. However, this study did show improved biochemical progression free survival during the entire follow‐up period, as well as improved metastasis‐free survival and improved overall survival. Current NCCN recommendations discourage the use of neoadjuvant ADT prior to RP unless enrolled on a clinical trial [15]. However, in the setting of limited resources, OR times, and RT treatment slots, such as during a global pandemic, proceeding with neoadjuvant ADT prior to definitive surgery is a safe option, with possible therapeutic benefits and low likelihood of detriment to the patient.

As noted previously, we did see a significant increase in the odds of patients with higher‐risk disease in the form of higher GG and HR/VHR receiving treatment during COVID in the adjusted models. While all patients should have input into their treatment decision process for prostate cancer, strategies such as active surveillance or ADT alone depending on risk and life expectancy remain potential options [15]. When resources and staffing are limited and risk of exposure to staff during a pandemic is present, there should be a trend toward treating patients with higher‐risk disease and potentially delaying treatment in lower‐risk disease, so long as oncologic outcomes are not compromised. This was recommended by multiple groups for treatment of prostate cancer during COVID‐19 and seen in practice [7, 12, 14, 17, 18]. Following these guidelines, oncologists must also ensure that patients are indeed getting appropriate and timely oncologic care. This discussion is relevant not only in the pandemic setting but also in the setting of staffing shortages. Currently in RT oncology, there is a national staffing shortage, as demonstrated by a survey through ASTRO [33]. Of the respondents, 93% reported staffing shortages of key clinical staff, including nursing, RT therapists, physicists, and dosimetrists. In fact, the American Registry of Radiologic Technologists released a white paper aimed at understanding and addressing these labor shortages in the field [34]. Longer‐term follow‐up with regard to treatment delays and outcomes is warranted when viewing care for prostate cancer patients, as well as patients with other malignancies.

The limitations of this study include its retrospective nature and single institutional experience. Likewise, in patients with prostate cancer, differences in outcome generally take years to accurately determine, thus limiting any potential therapeutic outcome conclusions generated from these data.

## 5. Conclusions

This study contributes to the growing body of literature discussing changes of prostate cancer management during the COVID‐19 pandemic. While there are multiple limitations to this study, we did find differences in longer TT, prolonged time from ADT initiation to RT, increased utilization of ADT for patients planned for surgery, and prioritizing treatment for higher‐risk disease. Analyzing potential effects on oncologic outcomes based on these findings is warranted, although this will require long‐term follow‐up. Overall, these findings may help inform oncologists on how to approach therapeutic options for prostate cancer patients during times of limited resources, such as during a pandemic.

## Ethics Statement

This study was approved under our institutional review board, and thus no patient consent was required to proceed with this study.

## Disclosure

The results from this study were presented at the 105^th^ Annual Meeting of the Proceedings of the American Radium Society.

## Conflicts of Interest

The authors declare no conflicts of interest.

## Author Contributions

Alexander Yaney and Jonathan E. Schoenhals contributed equally to this work.

## Funding

No grant support was used for this study.

## Supporting Information

Supporting Information Table 1 shows a multivariate analysis based on various patient factors using GG.

Supporting Information 2 shows further stratification of the outcomes described in this manuscript based on NCCN risk grouping of low‐risk disease and high‐risk disease.

## Supporting information


**Supporting Information** Additional supporting information can be found online in the Supporting Information section.

## Data Availability

The deidentified data utilized to generate results for this study are available from the corresponding author for reasonable requests.
