# Supplementary material for: Change in Clinical Management of Localized Prostate Cancer Patients at a Tertiary Medical Center as a Result of SARS‐CoV‐2 (COVID‐19)
Source: Prostate Cancer. 2026 Jan 15;2026:9996270. doi: 10.1155/proc/9996270 (PMC12808921; doi:10.1155/proc/9996270)
Supplement: Supplementary file 1 — Supporting Information Additional supporting information can be found online in the Supporting Information section. [file PROC-2026-9996270-s001.docx]

**Supplementary Table 1 Multivariable analysis of treatment modality and patient factors for COVID compared to pre-COVID**

| **Variable** | **Crude OR (95% CI), p-value** | **Adjusted OR (95% CI), p-value** ^1^ |
| --- | --- | --- |
| **Treatment**  Surgery  Definitive RT | Ref  1.42 (0.93-2.16), p=0.105 | Ref  1.45 (0.92-2.28), p=0.108 |
| **Age** | 0.99 (0.97-1.02), p=0.646 | 0.98 (0.95-1.01), p=0.107 |
| **Race**  White  Non-White (Black, Other) | Ref  1.14 (0.69-1.87), p=0.610 | Ref  0.96 (0.57-1.62), p=0.889 |
| **PSA** | 1.01 (1.00-1.02), p=0.069 | 1.01 (1.00-1.02), p=0.263 |
| **Grade Group**  1-3  4-5 | Ref  1.85 (1.21-2.82), p=**0.004** | Ref  1.76 (1.11-2.78), p=**0.016** |

^1^ Adjusted for treatment, age, race, PSA and Grade Group

Abbreviations: OR=Odds Ratio, CI=Confidence Interval

**Supplementary Table 2 Comparing various outcomes based on risk stratification for pre-COVID and COVID**

|  | **Risk Group** | | | | | | |
| --- | --- | --- | --- | --- | --- | --- | --- |
|  | **High Risk Disease^2^** | | |  | **Low Risk Disease^3^** | | |
|  | **Time Frame** | |  |  | **Time Frame** | |  |
|  | **Pre-COVID** | **COVID** | **P-value** |  | **Pre-COVID** | **COVID** | **P-value** |
| **Time from diagnosis to treatment (days)**¹ |  |  | 0.045 |  |  |  | 0.088 |
| Median (IQR) | 109 (81, 157) | 138.5 (87, 213) |  |  | 111 (83, 153.5) | 120 (89, 190) |  |
| Range | 37-512 | 41-369 |  |  | 26-745 | 0-349 |  |
|  |  |  |  |  |  |  |  |
| **Treatment**, n (%) |  |  | 0.109 |  |  |  | 0.554 |
| Surgery | 67 (67.00%) | 32 (54.24%) |  |  | 154 (70.32%) | 50 (66.67%) |  |
| Definitive RT | 33 (33.00%) | 27 (45.76%) |  |  | 65 (29.68%) | 25 (33.33%) |  |
|  |  |  |  |  |  |  |  |
| **ADT receipt prior to surgery**, n (%) |  |  | 0.004 |  |  |  | 0.059 |
| Yes | 1 (1.49%) | 6 (18.75%) |  |  | 0 (0.00%) | 2 (4.00%) |  |
| No | 66 (98.51%) | 26 (81.25%) |  |  | 154 (100.00%) | 48 (96.00%) |  |
|  |  |  |  |  |  |  |  |
| **Time from diagnosis to surgery (days)** |  |  | 0.373 |  |  |  | 0.505 |
| Median (IQR) | 95 (67, 118) | 83 (67.5, 105.5) |  |  | 109 (78, 142) | 105.5 (62, 159) |  |
| Range | 37-512 | 41-164 |  |  | 26-512 | 0-349 |  |
|  |  |  |  |  |  |  |  |
| **Time from diagnosis to definitive RT (days)** |  |  | 0.008 |  |  |  | 0.034 |
| Median (IQR) | 166 (126, 214) | 240 (180, 286) |  |  | 130 (90, 179) | 175 (109, 224) |  |
| Range | 68-375 | 83-369 |  |  | 28-745 | 72-345 |  |
|  |  |  |  |  |  |  |  |
| **Time from diagnosis to salvage RT (days)** |  |  | 0.255 |  |  |  | 0.014 |
| Median (IQR) | 140 (100, 161) | 146 (111, 199) |  |  | 107 (82, 131) | 143 (109, 201.5) |  |
| Range | 45-336 | 60-286 |  |  | 34-252 | 77-250 |  |
|  |  |  |  |  |  |  |  |
| **Time from ADT to definitive RT (days)** |  |  | 0.004 |  |  |  | 0.291 |
| Median (IQR) | 78.5 (70, 129) | 152 (111, 199) |  |  | 71 (48, 119) | 95 (76, 180) |  |
| Range | 40-266 | 48-341 |  |  | 30-350 | 69-253 |  |
|  |  |  |  |  |  |  |  |
| **Time from ADT to salvage RT (days)** |  |  | 0.083 |  |  |  | 0.421 |
| Median (IQR) | 75 (58, 113) | 91 (71, 117) |  |  | 62 (48, 87) | 69 (58, 105) |  |
| Range | 40, 304 | 32, 265 |  |  | -1, 213 | 21, 189 |  |

^1^ Measured among entire cohort (surgery, definitive RT and salvage RT)

^2^ Defined as HR and VHR

^3^ Defined as VLR, LR, FIR, and UIR
